# Pennsylvania Hospital—Surgical Wards—Service of Dr. Norris

**Published:** 1842-03-26

**Authors:** E. Hartshorne

**Affiliations:** Resident Surgeon


### CLINICAL REPORTS.

### *Pennsylvania Hospital—Surgical Wards—Service of Dr. Norris.*

By Dr. E. Hartshorne, Resident Surgeon.

*Fractured patella.*—Henry O'H., 60 years of age, feeble and emaciated, entered Nov. 11, with a transverse fracture of the left patella. The man stated that while walking in a cellar, he stumbled, and in falling struck his knee against the jutting end of a log of wood. He was unable to rise and immediately observed a depression in the knee-pan wide and deep enough to admit his thumb.

When brought into the ward, about eighteen hours after the accident, he appeared to be in great suffering; the joint had already become very tumid and tense, and so painful as to preclude any but a superficial examination into the condition of the parts. It was ascertained, however, that the fragments were at least an inch and a half if not two inches asunder.

The limb was extended, confined with a roller in this position on a back splint long enough to reach from just below the tuber ischii nearly to the heel, and elevated on an inclined plane, so as to flex the thigh on the pelvis—at the same time the body of the patient was propped up with pillows in order still more completely to secure the relaxation of the muscles, as well as to afford the man an easy recumbent position.

Owing to the great violence of the inflammation no forcible approximation of the fragments could be tolerated by the patient before the end of the first week. During this interval, the treatment by position was combined with the ordinary antiphlogistic regimen of rest, low diet, copious leeching, and saturnine lotions, rigidly maintained. On the eighth day the separated parts were brought within two or three lines of each other; and so maintained, in the following manner. Moderate and uniform compression of the limb having been effected by a roller carefully applied from the toes up to the middle of the thigh, the fractured surfaces were retained in their juxta-position by means of pads subjected to the pressure of a roller carried with some force around the knee and splint in two series of circular turns; the first of which operating on the upper margin of the patella, passed below the projecting ends of a cross-bar two inches wide, fixed transversely to the splints, at a point corresponding posteriorly to the tubercle of the tibia; the other series moved around the lower margin of the bone and above the projecting ends of the same cross piece. By this arrangement, the retraction of the upper fragment and the displacement of the lower were effectually counteracted without the constriction of the limb produced by the ordinary figure-of-eight bandage, especially when applied without the intervention of a splint and cross-bar.

In this case, as the sensibility in the injured joint did not rapidly abate even after the symptoms of more active inflammation had subsided, very little force was at first exerted on the retracting portion of the bone. This force was gradually increased in the course of the first three weeks, although it was not at any time very great, as the patient, being aged and of an irritable temperament, was intolerant of pain and bore the compression badly, while at no time did there appear in the muscles any strong disposition to contract. The lead-water dressing was continued at least fifteen days, until the heat of the part had nearly all disappeared.

Very firm and close union by ligament was found to be complete by the twenty-eighth day, the fragments being within the eighth of an inch of each other and entirely immovable. The patient was allowed to walk about on crutches with the splints still on, after confinement in bed about five weeks. In about ten days after leaving his bed he was directed to walk with the crutches alone, the splints being removed and a bandage only remaining. The knee was passively moved and well rubbed with soap liniment every day. Discharged January 29th, with the power of extending the limb very good, flexion being still difficult on account of the partial ankylosis arising from the treatment.

*Remarks.*—The present is the second case treated successfully in the last six months, with the apparatus here described. The first case was even more unfavourable than this. A young German was admitted July 10, having received a transverse fracture of the patella with a small wound of the soft parts in front slightly exposing the bone, caused by the kick of a horse. Severe inflammation of course soon invested the injured part to such an extent that position only was available in the management of the fracture until the acute symptoms had been subdued by active antiphlogistic measures. The compresses and bandage were first applied about the 20th day, at which time the external wound had nearly cicatrised. The fragments were brought very near together, and union by a dense ligament advanced very well. After having walked about with the splints on for two or three weeks, the man was discharged Sept. 8th, with a trifling separation of the bone and a very good use of the joint. The splint employed in these cases is that of Desault, modified by the addition of the cross piece. The bandages recommended by that authority, however, are omitted, the simpler bandage around the knee and splint being preferred. It was employed as the ordinary apparatus of the house twenty years ago, without being at the time considered original or peculiar. A writer in the London Lancet for 1835–6, about six years since, introduced it, however, as a novelty to British surgeons, and advocated its employment. Lonsdale, in his work, describes it, and attributes its contrivance to this writer. It certainly possesses simplicity and ease of application, without creating much discomfort to the patient, while it appears to answer all the indications extremely well.

